# Social and non-social autism symptoms and trait domains are genetically dissociable

**DOI:** 10.1038/s42003-019-0558-4

**Published:** 2019-09-03

**Authors:** Varun Warrier, Roberto Toro, Hyejung Won, Claire S. Leblond, Freddy Cliquet, Richard Delorme, Ward De Witte, Janita Bralten, Bhismadev Chakrabarti, Anders D. Børglum, Jakob Grove, Geert Poelmans, David A. Hinds, Thomas Bourgeron, Simon Baron-Cohen

**Affiliations:** 1 Autism Research Centre, Department of Psychiatry, University of Cambridge, Cambridgeshire, UK; 2 Human Genetics and Cognitive Functions, Institut Pasteur, UMR3571 CNRS, Université de Paris, Paris, France; 3 Department of Genetics and Neuroscience Center, University of North Carolina, Chapel Hill, NC 27599 USA; 4 Child and Adolescent Psychiatry Department, Robert Debré Hospital, Paris, France; 5 Department of Human Genetics, Radboud University Medical Center, Nijmegen, The Netherlands; 6 Donders Institute for Brain, Cognition and Behaviour, Radboud University, Nijmegen, The Netherlands; 7 Centre for Autism, School of Psychology and Clinical Language Sciences, University of Reading, Reading, UK; 8 The Lundbeck Foundation Initiative for Integrative Psychiatric Research, iPSYCH, Aarhus, Denmark; 9 Centre for Integrative Sequencing, iSEQ, Aarhus University, Aarhus, Denmark; 10 Department of Biomedicine - Human Genetics, Aarhus University, Aarhus, Denmark; 11 Bioinformatics Research Centre, Aarhus University, Aarhus, Denmark; 12 23andMe Inc., Mountain View, CA 94043 USA

**Keywords:** Autism spectrum disorders, Genetic predisposition to disease, Genome-wide association studies

## Abstract

The core diagnostic criteria for autism comprise two symptom domains – social and communication difficulties, and unusually repetitive and restricted behaviour, interests and activities. There is some evidence to suggest that these two domains are dissociable, though this hypothesis has not yet been tested using molecular genetics. We test this using a genome-wide association study (*N* = 51,564) of a non-social trait related to autism, systemising, defined as the drive to analyse and build systems. We demonstrate that systemising is heritable and genetically correlated with autism. In contrast, we do not identify significant genetic correlations between social autistic traits and systemising. Supporting this, polygenic scores for systemising are significantly and positively associated with restricted and repetitive behaviour but not with social difficulties in autistic individuals. These findings strongly suggest that the two core domains of autism are genetically dissociable, and point at how to fractionate the genetics of autism.

## Abstract (web summary)

Varun Warrier et al. report a genome-wide association study of systemising, a non-social trait associated with autism. They find 3 loci associated with systemising and show that this trait has no significant genetic correlations to social phenotypic measures, demonstrating that the social and non-social aspects of autism are genetically distinct.

## Introduction

The core diagnostic criteria of autism comprises two symptom domains: difficulties in social interactions and communication (the social domain), and unusually repetitive and restricted behaviour and stereotyped interests (the non-social domain)^1^. Multiple lines of evidence suggest that these two domains are dissociable^2,3^. First, factor and principal component analysis of autism and autistic traits have predominantly identified two factors—a social and a non-social factor^4–9^. Second, investigations of autistic traits in large cohorts have demonstrated a positive phenotypic correlation between different social traits and different non-social traits separately, but only a limited correlation between social and non-social traits^9–12^. Third, twin genetic correlations between social and non-social symptom domains in autism are low, although both social and non-social trait domains are highly heritable in neurotypical^13,14^ or autistic twins^15^. Fourth, difficulties in social and non-social domains can occur independently of each other^16,17^, which has been used to subgroup individuals on the spectrum based on the two domains^18^. This suggests that the genetic and phenotypic architecture of autism consists of at least two broadly dissociable domains. This has implications for genetic, biological, and clinical studies of autism, since most studies have investigated autism as if it is a unitary condition^3^. The idea that social and non-social symptom domains are dissociable is unsurprising given their very different nature, and very different underlying neurology and cognitive processes: one related to interpreting animate motion and mental states (theory of mind) and the other related to recognising inanimate objects, events or patterns (systemising)^3^. Nevertheless, a diagnosis of autism is only given when the social and non-social symptom domains cluster together.

However, to date, there has been limited molecular genetic evidence in support of this dissociability hypothesis, partly due to the limited large-scale research on the genetics of social and non-social domains. Most genetic research into the social and non-social domains has been primarily through linkage and genome-wide association studies (GWAS) in relatively small samples of autistic individuals and the general population (*N* < 5K)^19–25^. This has precluded a detailed molecular genetic investigation of the social and non-social domains associated with autism. Given currently available sample sizes with phenotypic information, investigating the genetics of the social and non-social domains in autistic individuals is difficult. However, several studies have demonstrated that the underlying liability for autism is normally distributed in the general population^26–29^. Factor analyses have failed to identify discontinuities between clinical autism and autistic traits in the general population^30^. Autistic traits are heritable^31–33^, are elevated in family members of autistic individuals compared to the general population^34,35^, and are transmitted intergenerationally^36,37^. Factor analysis of autistic traits measures have also identified two different factors in both the general population and autistic individuals—one linked to the social domain, and another linked to the non-social domain, mirroring the factor structure of clinical autism domains^6,9,30,38^. Studies have further demonstrated moderate to high shared genetics between the extremes of the liability distribution and the rest of the distribution^14,39–41^. One twin study investigated the bivariate genetic correlation between research and clinical autism diagnosis and autistic traits and identified high genetic correlations (0.7 < *r*_g_ < 0.89)^42^. Validating this, studies have identified modest shared genetics between autism and autistic traits^43–45^. Taken together, there is considerable evidence to suggest that autism represents the extreme end of the autistic traits continuum.

While a few studies have investigated the genetics of traits contributing to the social domains such as social and communications difficulties^19,44,45^, empathy^46^, and emotion recognition^47^, there have been limited studies investigating the genetics of the non-social domain^25,48^. Neither of these studies have replicably identified significant variants associated with the non-social domain, primarily because of the relatively modest sample sizes of the GWAS. An alternate approach is to investigate the genetics of non-social traits related to autism in the typical population, maximising the sample size. To better understand the genetics of a non-social trait related to autism, we investigate the genetics of systemising measured using a 75-item well validated, self-report measure called the Systemising Quotient-Revised (SQ-R) (see ‘Methods’ section). Systemising involves identifying input-operation–output (or if-and-then) relationships in order to analyse and build systems, and to understand the laws that govern specific systems^49^. The hyper-systemising theory of autism proposes that autistic individuals, on average, have superior attention to detail, and a stronger drive to systemise compared to individuals in the general population^49^. This has been validated in several studies^50,51^ including a recent study in more 650,000 individuals, including 36,000 autistic individuals^12^. Several lines of evidence suggest that autistic individuals have at least intact if not superior systemising. The idea was noted in the earliest papers describing autism by both Asperger^52^ and Kanner^53^, although these early papers do not use the term ‘systemising’ but instead comment on strong interests in pattern recognition, the need for order and predictability, excellent memory for facts, and a strong focus on objects and understanding how things work. Further, autistic adults, on average, score higher on the SQ-R compared to individuals in the general population^10,51^, a profile also observed in autistic children^54^. Several items in the SQ-R specifically measure circumscribed interests and insistence on sameness, two of the items mentioned in the DSM-5, and several of these items map onto items on the Autism Spectrum Quotient (AQ), a well validated measure of autistic traits^27^ (see Supplementary Note). Because systems follow rules, they repeat. A fascination with systems may thus manifest as unusually repetitive behaviour. And because systems depend on precise variables, a fascination with systems may also manifest as unusually narrow interests in autism.

The present study has two aims: first, to investigate the genetic architecture of a non-social trait linked to autism (systemising); and second, to investigate whether social and non-social traits related to autism, measured in the general population, are genetically dissociable.

## Results

### GWAS results

**Fig. 1:**
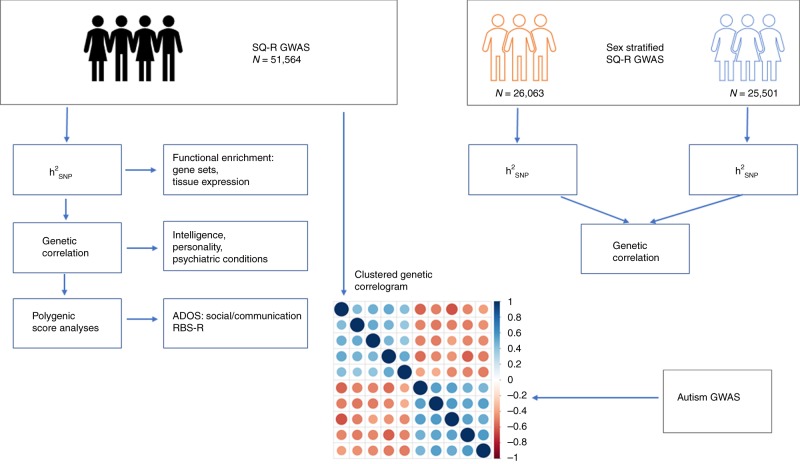
Schematic diagram of the study. We conducted a GWAS of the SQ-R (*N* = 51,564) and quantified SNP heritability $\left( {{{h}}_{{\mathrm{SNP}}}^2} \right)$, quantified genetic correlations with multiple phenotypes, and conducted polygenic score analyses. In addition, we conducted sex-stratified GWAS of the SQ-R, and investigated ${{h}}_{{\mathrm{SNP}}}^2$ within sex and genetic correlation between males and females. Finally, we investigated the clustering of all phenotypes that are genetically correlated with autism, and whether the social and the non-social phenotypes associated with autism are genetically correlated

We first conducted a GWAS of systemising (*N* = 51,564) measured using the SQ-R. Following this, and using data from GWAS of social traits genetically correlated with autism (GWAS of self-reported empathy (*N* = 46,861)^46^, and GWAS of social relationship satisfaction^55^ measured using friendship (*N*_effective_ = 164,112) and family relationship (*N*_effective_ = 158,116) satisfaction scales), we investigated whether the social and non-social traits related to autism are genetically dissociable in the general population. A flow chart of the study design is provided in Fig. 1.

**Fig. 2:**
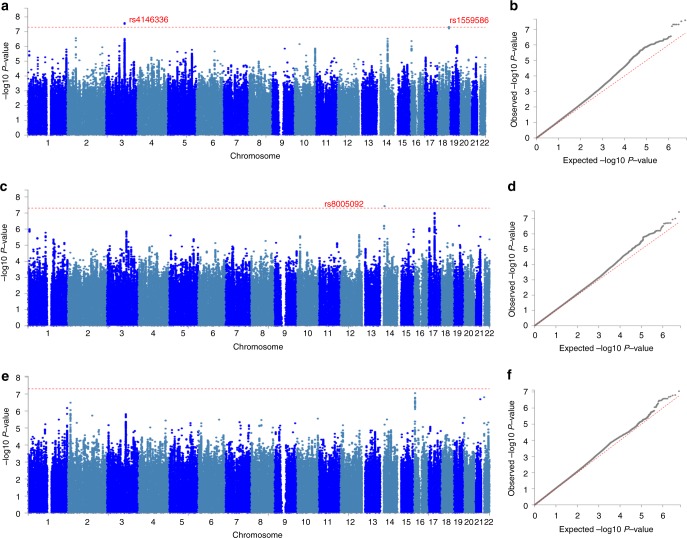
Manhattan and QQ-plots for the three GWAS. Manhattan plot for the three SQ-R GWAS: non-stratified (**a**), males-only (**c**), females-only (**e**). Significant SNPs are highlighted in red. QQ-plots for the three SQ-R GWAS: non-stratified (**b**), males-only (**d**), females-only (**f**). SQ-R non-stratified (*N* = 51,564): *λ*_GC_ = 1.10, LDSR intercept = 0.99, SQ-R males-only (*N* = 26,063): *λ*_GC_ = 1.06, LDSR intercept = 0.99, SQ-R females-only (*N* = 25,501): *λ*_GC_ = 1.05, LDSR intercept = 1.01

Systemising was measured in the 23andMe sample (*N* = 51,564) using scores from the SQ-R^10^. Scores on the SQ-R were normally distributed, with a mean of 71 ± 21 out of 150. As hypothesised based on previous research^10,12,51^, males (76.5 ± 20), on average, scored higher than females (65.4 ± 20.6) (*P* < 0.001, Cohen’s *d* = 0.54, Supplementary Fig. 1). Given the significant sex differences in scores, we conducted a non-stratified and sex-stratified GWAS for the SQ-R. Genome-wide association analyses identified three significant loci (Fig. 2, Supplementary Data 1 and Supplementary Fig. 2). Two of these were significant in the non-stratified GWAS: rs4146336 on chromosome 3 (*P* = 2.58 × 10^−8^) and rs1559586 on chromosome 18 (*P* = 4.78 × 10^−8^). The third significant locus was in the males-only GWAS (rs8005092 on chromosome 14, *P* = 3.74 × 10^−8^). rs8005092 and rs1559586 lie in regions of high genetic recombination. Linkage-disequilibrium score regression (LDSR) intercept suggested that there was minimal inflation due to population stratification (Fig. 2). Fine-mapping of the three regions identified 14 credible SNPs (see ‘Methods’ section). None of the SNPs overlapped with fetal brain eQTL. However, two of these SNPs mapped onto two genes—*LSAMP* and *PTMAP8*, both of chromosome 3—using chromatin interaction data in the fetal brain. Of these, LSAMP is a neuronal adhesion molecule in the limbic system of the developing brain. In addition, gene-based analysis identified four significant genes *SDCCAG8*, *ZSWIM6*, *ZNF574*, and *FUT8* (Supplementary Data 2). Of these, mutations in *ZSWIM6* cause a neurodevelopmental disorder with, in some cases, co-morbid autism and unusually repetitive movements and behaviour^56^. As supporting analyses, we investigated the direction of effect for all independent SNPs with *P* < 1 × 10^−6^ in the non-stratified SQ-R GWAS in GWAS of autism^57^, educational attainment^58^, and cognitive aptitude^59^. Five out of six SNPs tested had concordant effect direction in the GWAS for educational attainment and GWAS for cognitive aptitude (*P* = 0.21, two-sided binomial sign test for each comparison). Similarly, four out of five SNPs tested had concordant effect direction in the GWAS for autism (Supplementary Data 3a) (*P* = 0.37, two-sided binomial sign test). For these three phenotypes, we additionally assessed effect direction concordance using binomial sign test at less stringent *P*-value thresholds in the SQ-R GWAS, after LD-based clumping (*P* < 1, 0.5, 0.1 and 1 × 10^−4^). Binomial sign test was statistically significant at three of the four *P*-value thresholds (*P* = 1, 0.5 and 0.1) for all three phenotypes but not statistically significant at *P* = 1E−4, presumably due to the low statistical power (Supplementary Data 3b). In addition, we tested effect direction concordance (*P* < 1 × 10^−6^) in a GWAS (*N* = 1981) of ‘insistence on sameness’, a phenotype similar to systemising (see ‘Methods’ section). Four out of five SNPs had a concordant effect direction including the two SNPs with *P* < 5 × 10^−8^ in the non-stratified SQ-R GWAS (*P* = 0.37, two-sided binomial sign test).

### Genetic correlation between the SQ-R and other phenotypes

Additive SNP-based heritability $\left( {{{h}}_{{\mathrm{SNP}}}^2} \right)$ calculated using LDSR was 0.12 ± 0.012 for the SQ-R (*P* = 1.2 × 10^−20^). Despite small but significant sex differences in the SQ-R scores, there was no significant difference in ${{h}}_{{\mathrm{SNP}}}^2$ between males and females (*P* = 0.34) (Supplementary Fig. 3 and Supplementary Data 4), which was strengthened by the high genetic correlation between males and females (1 ± 0.17; *P* = 3.91 × 10^−10^), suggesting a similar polygenic architecture between sexes. The per-SNP effect for the most significant SNPs was small, suggesting a highly polygenic architecture (*R*^2^ = 0.001–0.0002%, after correcting for winner’s curse, Supplementary Data 5).

Partitioned heritability for functional categories identified significant enrichment for evolutionary conserved regions, transcription start sites, fetal DNase hyper-sensitivity sites, and H3 lysine 27 acetylation (H3K27ac), suggesting a prominent role for regulatory and conserved genomic regions in systemising (Supplementary Data 6). Partitioning heritability based on tissue-specific active chromatin marks identified a significant enrichment for brain specific chromatin signatures. Notably, this enrichment was significant in both adult and fetal brain specific active chromatin marks (Supplementary Data 7 and Supplementary Fig. 4). Enrichment for genes expressed in the brain was high but failed to reach statistical significance after correcting for the multiple tests conducted (Supplementary Fig. 5 and Supplementary Data 8).

**Fig. 3:**
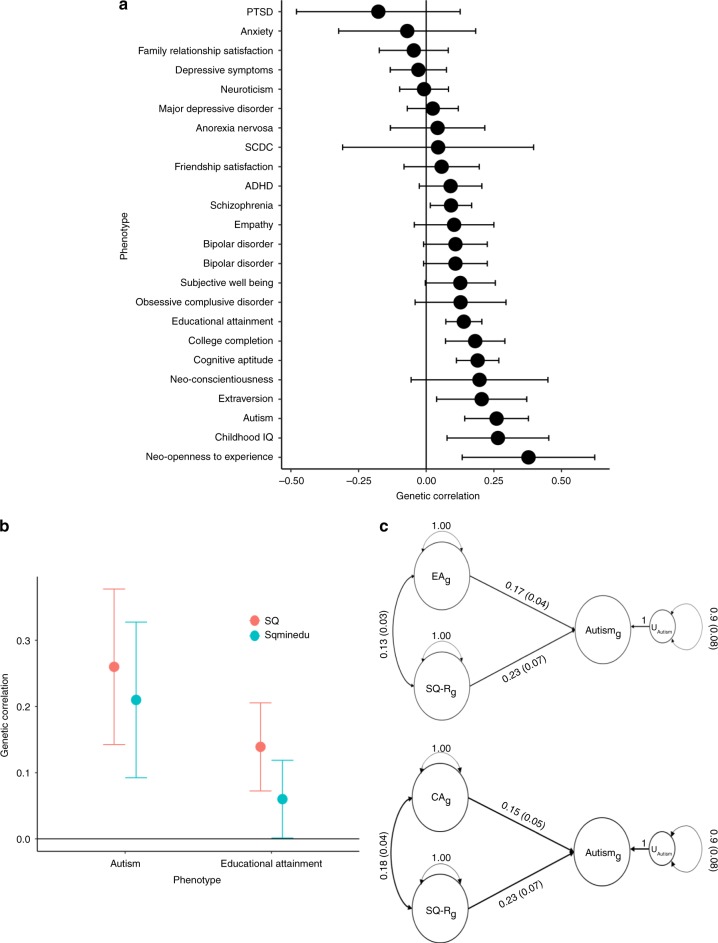
Genetic correlation between the SQ-R and other phenotypes, and GWIS and GSEM estimates between SQ, educational attainment and cognitive aptitude. **a** Genetic correlations between the SQ-R and multiple other phenotypes provided. The bars represent 95% confidence intervals. Sample sizes and PMID are provided in Supplementary Data 9. The following genetic correlations were significant after Bonferroni correction: autism (*r*_g_ = 0.26 ± 0.06; *P* = 3.35 × 10^−5^, *N* = 46,350), years of schooling (*r*_g_ = 0.13 ± 0.03; *P* = 4.73 × 10^−5^, *N* = 293,723), college completion (*r*_g_ = 0.18 ± 0.05; *P* = 1.30 × 10^−3^, *N* = 95,427), and cognitive aptitude (*r*_g_ = 0.19 ± 0.04; *P* = 2.35 × 10^−5^, *N* = 78,308). **b** Results of the GWIS analysis. Red lines represent genetic correlation with the SQ-R, blue lines represent genetic correlations with the SQ-R independent of the genetic effects of educational attainment. The bars represent 95% confidence intervals. **c** Path diagrams providing the results of the standardised SEM models to investigate whether the SQ-R is genetically correlated with autism independent of the genetic effects of cognitive aptitude (CA_g_) and educational attainment (EA_g_). GWIS genome-wide inferred statistics, GSEM genomic structural equation modelling

We identified a significant positive genetic correlation between the SQ-R and autism as well as measures of intelligence (cognitive aptitude and educational attainment) (Supplementary Data 9 and Fig. 3a). Of all the psychiatric conditions tested (see ‘Methods’ section), SQ-R was only significantly genetically correlated with autism (*r*_g_ = 0.26 ± 0.06; *P* = 3.35 × 10^−5^), demonstrating the relative specificity of the correlation of the SQ-R to autism. Notably, the absolute magnitude of the genetic correlation between autism and the SQ-R is similar to the genetic correlation between autism and self-reported empathy (measured using the Empathy Quotient (EQ^60^): *r*_g_ = −0.27 ± 0.07) and scores on the Social and Communication Disorders Checklist (SCDC^61^): *r*_g_ = 0.27 ± 0.13). Controlling for the genetic effects of educational attainment on the SQ-R GWAS using genome-wide inferred statistics (GWIS) (see ‘Methods’ section) attenuated the genetic correlation with autism only modestly, suggesting that the SQ-R scores are genetically correlated with autism independently of the genetic effects of education (Fig. 3b and Supplementary Data 10). We validated this using genomic structural equation modelling (GSEM) (see ‘Methods’ section) using both educational attainment and cognitive aptitude (Fig. 3c). Further, the SQ-R was not genetically correlated with any of the social measures related to autism—friendship and family relationship satisfaction, scores on a self-report measure of empathy (the EQ), and the scores on the Social and Communication Disorders Checklist (SCDC), which is a measure of social and communication difficulties (see Supplementary Note for how these traits map onto social domains in autism). Estimates of genetic correlations between SQ-R scores and the various social traits are also small, suggesting that there is limited shared genetics between social autism traits and the SQ-R.

### Genetic correlations between social/non-social traits and psychiatric conditions

**Fig. 4:**
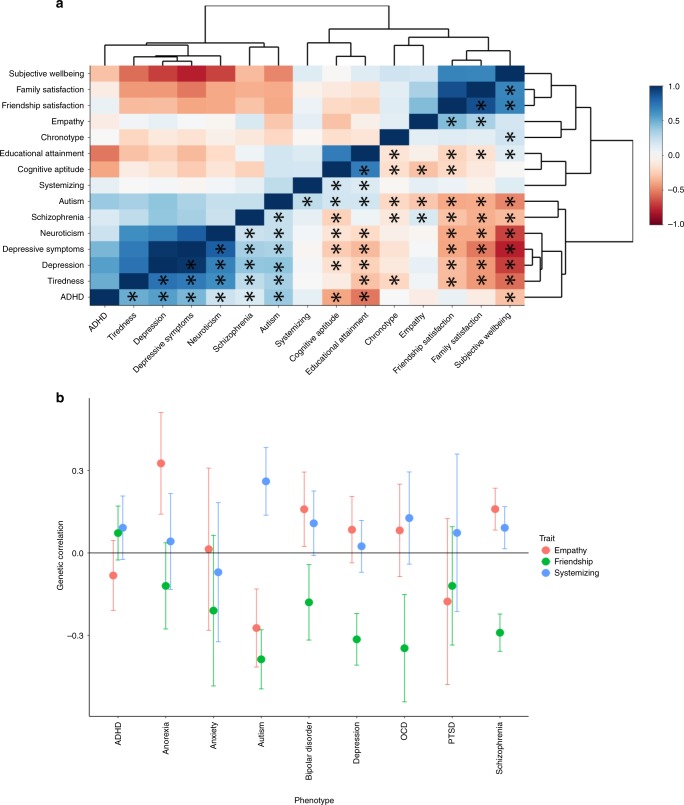
Genetic correlogram of autism and related traits, and genetic correlations between social and non-social traits and multiple psychiatric conditions. **a** Correlogram of genetic correlations between all phenotypes that are genetically correlated with autism. Please note the upper and lower triangle are identical. Asterisk (provided only in the lower triangle) represents significant correlations after Bonferroni correction. Genetic correlations have been clustered using hierarchical clustering. Colour provides the magnitude of genetic correlation. **b** Genetic correlation between empathy, friendship satisfaction, and systemising with nine psychiatric conditions. Only autism was significantly genetically correlated with all three phenotypes. Full results are present in Supplementary Data 11

To understand the genetic relationship between the SQ-R and autism in a broader context, we evaluated the genetic correlations between multiple phenotypes with evidence of significant genetic correlation with autism (15 phenotypes in total, see ‘Methods’ section for a list of phenotypes included). Clustering highlighted three broad clusters: a social cluster, a psychiatric cluster, and an intelligence cluster (Fig. 4a and Supplementary Tables 11 and 12). The SQ-R clusters closely with measures of intelligence, but while educational attainment and cognitive aptitude are significantly genetically correlated with multiple social traits and psychiatric conditions, the SQ-R is only genetically correlated with autism.

Given that the two major domains of autism as identified by the DSM-5 are persistent difficulties in social interaction and communication and unusually restrictive, stereotyped, and repetitive interests^1^, we hypothesised that the combination of significant negative genetic correlation with social traits (friendship satisfaction and empathy) and significant positive genetic correlation with SQ-R would be uniquely associated with autism (see ‘Methods’ section). Indeed, across the nine psychiatric conditions for which we had summary GWAS statistics, this combination was uniquely observed for autism (Fig. 4b, Supplementary Data 13).

### Validation in additional cohorts

Given that our current analysis focussed on the general population, we sought to investigate whether polygenic scores from the SQ-R were associated with social and non-social autism domains in 2221 autistic individuals from the Simons simplex collection (see ‘Methods’ section). We hypothesised that SQ-R may be significantly associated with the non-social domain in autism, but not associated with the social domain in autism. Polygenic scores for SQ-R were significantly associated with scores on the Repetitive Behaviour Scale-Revised (RBS-R) (beta = 0.052 ± 0.02, *P* = 0.013), but not on the social and communication subscale of ADOS-G (beta = −0.00099 ± 0.018, *P* = 0.95) after adjusting for multiple test (Bonferroni alpha = 0.025). We validated this in 426 additional individuals of which 401 had a diagnosis of autism with RBS-R scores from the EU-AIMS LEAP, AGRE, and Paris cohorts. Here, we identified a concordant effect direction for polygenic scores of the SQ-R (beta = 0.02 ± 0.05, *P* = 0.65), although the results were not significant potentially due to the small sample size. Inverse-variance meta-analysis of the discovery and the validation cohorts marginally improved the significance of the association (beta = 0.047 ± 0.018, *P* = 0.010), and the results remained statistically significant (Bonferroni alpha = 0.025). In a separate sample of 475 autistic individuals from the AGRE cohort, polygenic scores for the SQ-R were not associated with the social and communication subscale of ADOS-G (beta = −0.046 ± 0.04, *P* = 0.24). Meta-analysis of the two cohorts did not produce a statistically significant result (beta = −0.008 ± 0.016, *P* = 0.60) (see Power calculations in the Supplementary Note). We note that the lack of association between the polygenic scores for the SQ-R and the ADOS-G social and communication subscale is not indicative of absence of shared genetics, but rather indicative of lower shared genetics between the SQ-R and the ADOS-G social and communication subscale than that between the RBS-R and the SQ-R.

Finally, to further validate the results in autistic individuals, we conducted bivariate genetic correlations on scores on the RBS-R and the ADOS-G social and communication subscale in 2989 individuals from the SSC, AGRE, EU-AIMS LEAP and Paris cohorts (2964 autistic individuals). Both the RBS-R $\left( {{{h}}_{{\mathrm{SNP}}}^2 = 0.11 \pm 0.11,{{P}} = 0.15} \right)$ and the ADOS-G social and communication subscale $\left( {{{h}}_{{\mathrm{SNP}}}^2 = 0.26 \pm 0.10,{{P}} = 0.004} \right)$ had modest ${{h}}_{{\mathrm{SNP}}}^2$, though only the latter was statistically significant. We identified a small genetic correlation (*r*_g_ = 0.15 ± 0.46, *P* = 0.74), which was not statistically different from 0. Given the small sample size, the genetic correlation is unlikely to be statistically significant. However, the effect was small and statistically less than 1 (*P* = 0.034, one-tailed *t*-test).

## Discussion

Here we present, to our knowledge, the largest GWAS of a non-social trait related to autism in the general population—systemising, measured using the SQ-R. We demonstrate that systemising is heritable and genetically correlated with autism. Associated loci are enriched in genomic regions containing brain chromatin signatures and we identify three genome-wide significant loci, but these must be replicated in an independent cohort. Despite the modest sample size, our GWAS is well-powered to investigate genetic correlations between various phenotypes including social traits related to autism, as the *Z*-score of the ${{h}}_{{\mathrm{SNP}}}^2$ is above the recommended threshold of four^62^. We identify high sign concordance of the top SNPs in genetically correlated traits, enrichment for active chromatin marks in fetal and adult brain, and significant polygenic score association with the RBS-R. Polygenic score analysis suggests that the shared genetics between systemising and the non-social domain of autism is considerably higher than the shared genetics between systemising and the social domain of autism. In addition, using a smaller sample of autistic individuals, we provide preliminary evidence that the social and non-social domains in autistic individuals have low shared genetics. Our results highlight the need to collect deeper clinical and cognitive information in autistic individuals to better understand the phenotypic heterogeneity in autism.

Most studies model autism and autistic traits as a single phenotype. This has likely arisen because of the difficulties in recruiting and phenotyping sufficient numbers of autistic people. Our study suggests that both in the general population and in autistic individuals, social and non-social autistic traits and symptom domains are genetically dissociable. This may to some extent explain why, compared to GWAS of other psychiatric conditions of roughly similar sample sizes^57,63–65^, the largest GWAS of autism to date has identified fewer loci. One possible explanation is statistical signal-attenuation because of the underlying heterogeneity. However, this does not necessarily suggest that systemising, or the other individual trait domains are less complex. For instance, we observe similar ${{h}}_{{\mathrm{SNP}}}^2$ for SQ-R, self-reported empathy^46^, and the largest and most recent GWAS of autism^57^.

It is important to investigate whether these domains are dissociable in a larger cohort of autistic individuals and identify potential convergence of the two domains in gene expression networks in the developing brain. Our results confirm the need to rethink our understanding of autism as existing along a single dimension^3,66^. We hypothesise that the dissociation of the two domains will extend to other research modalities in studies of autism and autistic traits. It is important to note that, while our results demonstrate two broadly dissociable autistic trait domains in the general population and in autistic individuals, more research is needed to identify other potentially dissociable domains and to investigate whether this dissociability is driven by different designs of phenotypic instruments (e.g. self-report vs informant report). For example, our research does not make a distinction between communication and social interaction abilities, or between sensory difficulties and repetitive behaviours, and future molecular genetic studies may identify varying levels of overlap between these domains. The same principle applies to other research modalities (neuroimaging, cognitive studies, hormonal assays, etc.) investigating the biology of autism and autistic traits. These different symptom domains of autism may contribute to different co-morbidities. Our results identify shared genetics between the social traits related to autism and psychiatric conditions such as schizophrenia and depression, but limited shared genetics between the SQ-R and these conditions.

## Methods

### Participants

The current study included participants from 23andMe (primary GWAS - SQ-R), from ALSPAC (GWAS of scores on the Social and Communication Disorders Checklist (SCDC)) and autistic individuals from the Simons Simplex Collection (SSC), the Autism Genetic Resource Exchange (AGRE), and the EU-AIMS LEAP and PARIS cohorts.

### 23andMe participants

Research participants in the GWAS of the SQ-R were from 23andMe and are described in detail elsewhere^67,68^. All participants provided informed consent and answered surveys online according to a human subjects’ research protocol, which was reviewed and approved by Ethical & Independent Review Services, an external AAHRPP-accredited private institutional review board (http://www.eandireview.com). All participants completed the online version of the SQ-R on the 23andMe participant portal. Only participants who were primarily of European ancestry (97% European Ancestry) were selected for the analysis using existing methods^69^. Unrelated individuals were selected using a segmental identity-by-descent algorithm^70^. A total of 51,564 participants completed the SQ-R (males = 26,063, and females = 25,501).

### ALSPAC participants

ALSPAC is a longitudinal cohort which recruited pregnant mothers in the Avon region of the UK. The ALSPAC cohort comprises 14,541 initial pregnancies from women in Avon resulting in a total of 13,988 children who were alive at 1 year of age. Children were enrolled in additional phases, described in greater detail elsewhere^71^. This study received ethical approval from the ALSPAC Law-and-Ethics Committee, and the Cambridge Human Biology Research Ethics Committee. Written informed consent was obtained from parent or a responsible legal guardian for the child to participate. Assent was obtained from the child participants where possible. We conducted a GWAS of scores on the SCDC in 5,421 individuals from ALSPAC.

### Other cohorts

We included data from four cohorts to conduct polygenic score and bivariate genetic correlation analyses. The SSC (*n* = 2221 unrelated autistic individuals) consists of simplex autistic families, and are described elsewhere^72^. The AGRE cohort (*n* = 482 unrelated autistic individuals) consists of multiplex autism families, details of which are provided elsewhere^73^. In addition, we included 401 individuals (including 25 neurotypical individuals) from the EU-AIMS LEAP^74^ and Paris^75^ cohorts. Across all cohorts, we included only unrelated individuals, who were predominantly of European Ancestry as defined by genetic principal components (5 SD deviations above or below the mean of PC1 and PC2 from the HapMap CEU population).

Additionally, we also included data from 1981 unrelated individuals (1000 males, 981 females) from the Nijmegen Biomedical Study (NBS) to provide support for the independent SNPs with *P* < 1 × 10^−6^ in the non-stratified GWAS. Participants were asked the question: ‘It upsets me if my daily routine is disturbed’, which is related to a non-social domain of autism, and is similar to an item in the Autism Spectrum Quotient. Further information including genotyping and quality control is provided elsewhere^43^. Genetic association for the top SNPs were conducted using age, sex, and the first five genetic principal components as covariates using linear regression.

### Phenotypes

The primary phenotype for this study is the SQ-R, which was used to conduct a GWAS in participants from 23andMe. The SQ-R is self-report measure of systemising drive, or interest in rule-based patterns^10^. The SQ-R taps a variety of domains of systemising, such as interest in mechanical (e.g. car engines), abstract (e.g. mathematics), natural (e.g. the weather), motor (e.g. knitting), and collectible (e.g. stamp collecting) systems. There are 75 items on the SQ-R, with a maximum score of 150 and a minimum score of 0. Scores on the test are normally distributed^10^. The SQ-R has good cross-cultural stability and good psychometric properties with Cronbach’s alpha ranging from 0.79 to 0.94 in different studies^76^. Test–retest reliability available in a Dutch sample indicated high reliability of 0.79 (Pearson correlation)^76^. This was supported by another study in 4058 individuals which identified high internal cohesion^77^. Exploratory followed by confirmatory factor analysis using Rasch modelling suggests that the SQ-R is unidimensional^77^. A sex difference has been observed in multiple studies with males, on average, scoring significantly higher than females^10,51^. Criterion validity shows that the SQ-R has a modest but significant correlation with the mental rotation test (*r* = 0.25, *P* = 0.013), as well as its subscales^78^. Autistic individuals, on average, score higher on the SQ-R in multiple different studies^10,51,79^. Further, the SQ-R also predicts autistic traits, with a combination of the SQ-R and the Empathy Quotient predicting as much as 75% of the variance on the autism spectrum quotient, a measure of autistic traits^10^. The SQ-R has been validated using a short form in a very large population of 600,000 controls and 36,000 autistic individuals^12^.

In addition, we used the following secondary phenotypes: SCDC in ALSPAC, ADOS-G social and communication scores and the RBS-R in the other cohorts. We also used a single question which is a measure of ‘insistence on sameness’ in the NBS cohort.

The SCDC is a questionnaire that measures difficulties in verbal and nonverbal communication, and social interaction including reciprocal social interaction^61^. The questionnaire consists of 12 questions, with scores ranging from 0 to 24, and with higher scores reflecting difficulties in social interaction and communication. The SCDC has good internal consistency (0.93) and good test–retest reliability (0.81)^61^. The SCDC has reasonable specificity and sensitivity in distinguishing autistic from control individuals^80^. Previous research has demonstrated that the SCDC is genetically correlated with autism^44,45,57^. We conducted a GWAS of SCDC to investigate whether it is genetically correlated with SQ-R in this study. We used mother-reported SCDC scores on children aged 8. Although SCDC has been measured at different ages in the ALSPAC cohort, we chose SCDC scores measured at age 8 as this has the largest sample size and has high ${{h}}_{{\mathrm{SNP}}}^2$^19^ (*h*^2^ = 0.24 ± 0.07).

We chose two measures of social and non-social traits in autistic individuals. For the social trait, we used the social and communication domain scores from the ADOS-G, a widely used instrument for diagnosing and assessing autism in four cohorts (SSC, AGRE, EU-AIMS LEAP, and Paris). Participants completed one of the following ADOS-G modules^81^: 1 (used for children with little or no phrase speech), 2 (for children with non-fluent speech), 3 (verbally fluent children), and 4 (verbally fluent adolescents and adults). For this study, we used the raw totals of the scores from the social domain and the communication domain, combined. Scores for all four modules range from 0 to 24. The ADOS-G has high overall internal consistency, and high test–retest reliability for the social and communication subscales^81^. The choice for combining the social and communication domain scores were informed by factor analysis which suggested that the two domains contribute to one underlying factor^82^.

In contrast to the social and communication domain, the restricted and repetitive behaviour domain of the ADOS-G has poor test–retest reliability (*r* < 0.6) and a smaller range of scores (0–8) as it captures fewer repetitive and restrictive behaviour^81^. Hence, for this study, we used sores on the RBS-R^83^. The RBS-R is a measure developed to specifically measure restricted and repetitive behaviours in autistic individuals and captures stereotyped, self-injurious, sameness, compulsive, ritualistic, and restricted behaviour^84^, and has high inter-rater reliability and internal consistency^84^. The RBS-R comprises 43 questions with scores ranging from 0 to 3 for each item based on a Likert scale.

‘Insistence on sameness’ in the NBS cohort was measured using a single item: ‘It upsets me if my daily routine is disturbed’. This is related to a non-social domain of autism, and is again similar to an item in the Autism Spectrum Quotient. Participants were asked to indicate on a 4-point Likert scale ‘definitely agree’, ‘slightly agree’, ‘slightly disagree’, ‘definitely disagree’.

### Genotyping, imputation, and quality control and genetic association in the 23andMe cohort

Details of genotyping, imputation and quality control in the 23andMe cohort are provided elsewhere^47^. Briefly, unrelated participants were included if they had a call rate of >98.5%, and were of primarily European ancestry (97% European ancestry). A total of 1,030,430 SNPs (including InDels) were genotyped or imputed. SNPs were excluded if: they failed the Hardy–Weinberg equilibrium test at *P* < 10^−20^; had a genotype rate of <90%; they failed the parent-offspring transmission test using trio data in the larger 23andMe research participant database; or if allele frequencies were significantly different from the European 1000 Genomes reference data (χ^2^ test, *P* < 10^−20^). Phasing was conducted using Beagle (version 3.3.1)^85^ in batches of 8000–9000 individuals. This was followed by imputation against all-ethnicity 1000 Genomes haplotypes (excluding monomorphic and singleton sites) using Minimac2^86^. Genetic association analyses were restricted to SNPs with a minor-allele frequency > 1%. After quality control, 9,955,952 SNPs (imputed and genotyped) were included in the GWAS.

Our primary analysis was an additive model of genetic effects and was conducted using a linear regression with age, sex, and the first five ancestry principal components included as covariates. In addition, given the modest sex difference, we also conducted sex-stratified analyses. SNPs were considered significant at a genome-wide threshold of *P* < 5 × 10^−8^. Leading SNPs were identified after LD-pruning using Plink (*r*^2^ > 0.8). Winner’s curse correction was conducted using an FDR-based shrinking^87^.

We calculated variance explained by first standardising the regression estimates and then squaring the estimates. This is equivalent to:

$$R^2 = \hat B_j^2\frac{{2\left( {{\mathrm{MAF}}_j} \right)(1 - {\mathrm{MAF}}_j)}}{{\sigma _y^2}},$$

where *R*^2^ is the proportion of variance explained for SNP_*j*_. $\hat B_j^2$ is the non-standardised regression coefficient, MAF is the minor-allele frequency for SNP_*j*_, and $\sigma _y^2$ is the variance of SQ. Further details of this formula are provided in the Supplementary Note.

### Genotyping, imputation, and quality control and genetic association in the ALSPAC

The SCDC^61^ scores were calculated from children of the 90 s (ALSPAC cohort)^71^, in children aged 8. In total, SCDC scores were available on *N* = 7,825 children. From this, we removed individuals for whom complete SCDC scores were not available. After excluding related individuals and individuals with no genetic data, data was available on a total of *N* = 5,421 unrelated individuals.

Participants were genotyped using the Illumina® HumanHap550 quad chip by Sample Logistics and Genotyping Facilities at the Wellcome Sanger Institute and LabCorp (Laboratory Corportation of America) using support from 23andMe. Individuals were excluded based on gender mismatches, high missingness (>3%), and disproportionate heterozygosity. We restricted subsequent analyses to individuals of European descent (CEU), which were identified by multi-dimensional scaling analysis and compared with Hapmap II (release 22). Individuals were also removed if cryptic relatedness, assessed using identity by descent, was >0.1. Genotyped SNPs were filtered out if they had >5% missingness, violated Hardy–Weinberg equilibrium (*P* < 1 × 10^−6^), and had a minor-allele frequency < 1%, resulting in a total of 526,688 genotyped SNPs. Haplotypes were estimated using data from mothers and children using ShapeIT (v2.r644)^88^. Imputation was performed using Impute2 V2.2.2^89^ against the 1000 genomes reference panel (Phase 1, Version 3). Imputed SNPs were excluded from all further analyses if they had a minor-allele frequency < 1% and info < 0.8. After quality control, there were 8,282,911 genotyped and imputed SNPs that were included in subsequent analyses.

Dosage data from BGEN files were converted using hard-calls, with calls with uncertainty > 0.1 treated as missing data. Post-imputation, we excluded SNPs that deviated from Hardy–Weinberg equilibrium (*P* < 1 × 10^−6^), with minor-allele frequency < 0.01 and missing call rates > 2%. We further excluded individuals with genotype missing rates > 5%. The SCDC score was not normally distributed so we log-transformed the scores and ran regression analyses using the first two ancestry principal components and sex as the covariates using Plink 2.0 (ref. ^90^).

The log-transformed SCDC scores (henceforth, SCDC scores) had a modest but significant ${{h}}_{{\mathrm{SNP}}}^2$ as quantified using LDSR $\left( {{{h}}_{{\mathrm{SNP}}}^2 = 0.12 \pm 0.05} \right)$. LDSR intercept (0.99) suggested that there was no inflation in GWAS estimates due to population stratification. The *λ*_GC_ was 1.013. We replicated the previously identified genetic correlation with autism^57^ (constrained intercept) using our SCDC GWAS (*r*_g_ = 0.45 ± 0.18, *P* = 0.01). In addition, we also identified a negative genetic correlation between educational attainment^58^ and SCDC (*r*_g_ = −0.30 ± 0.11, *P* = 0.007).

### Genomic inflation factor, heritability, and functional enrichment for the SQ-R GWAS

LDSR^91,92^ was used to calculate for inflation in test statistics due to unaccounted population stratification. Heritability was calculated using LDSR using the north-west European LD scores. Difference in heritability between males and females was quantified using:

$$Z = \frac{{h_{{\mathrm{males}}}^2 - h_{{\mathrm{females}}}^2}}{{\sqrt {{\mathrm{SE}}_{{\mathrm{males}}}^2 + {\mathrm{SE}}_{{\mathrm{females}}}^2} }},$$

where *Z* is the *Z*-score for the difference in heritability for a trait, $\left( {{{h}}_{{\mathrm{males}}}^2 - {{h}}_{{\mathrm{females}}}^2} \right)$ is the difference ${{h}}_{{\mathrm{SNP}}}^2$ estimate in males and females, and SE is the standard errors for heritability. Two-tailed *P*-values were calculated and reported as significant if *P* < 0.05.

For the primary GWAS (non-stratified analyses), we conducted functional annotation using FUMA^93^. We restricted our analyses to the non-stratified analyses due to the high genetic correlation between the sexes and the low statistical power of the sex-stratified GWAS. We conducted gene-based association analyses using MAGMA^94^ within FUMA and report significant genes after using a stringent Bonferroni corrected *P* < 0.05. In addition, we conducted enrichment for tissue specific expression and pathway analyses within FUMA. For the significant SNPs, we investigated enrichment for eQTLs using brain tissues in the BRAINEAC and GTEx^95^ database within FUMA. We further conducted partitioned heritability for tissue-specific active chromatin marks and baseline functional categories using extended methods in LDSR^96^.

### Hi-C-based annotations of fine mapped loci

We fine mapped three genome-wide significant loci (index SNPs: rs4146336 and rs1559586 or SQ; rs8005092 for SQ-R males) to obtain credible SNPs. First, we selected SNPs with *P* < 0.01 that are located in the LD region (*r*^2^ > 0.6) with an index SNP. LD structure within a locus was constructed by calculating correlations between SNPs within a locus (1KG v20130502). CAVIAR^97^ was then applied to the summary association statistics and LD structure for each index SNP to generate potentially causal (credible) SNPs with a posterior probability of 0.95. In total, we identified 14 credible SNPs from the three GWS loci.

For each locus, candidate genes were identified by mapping credible SNPs based on physical interactions in foetal brain as previously described^98^. One locus (index SNP rs4146336) was mapped to two genes, *LSAMP* and *PTMAP8*, indicating that two credible SNPs (rs13066948 and rs11713893) located in this locus physically interact with these genes.

### Genetic correlation

For all phenotypes, we performed genetic correlation without constraining the intercept using LDSR. We identified significant genetic correlations using a Bonferroni adjusted *P*-value < 0.05. For the primary genetic correlation analysis with SQ-R, we included psychiatric conditions^57,63,99–102^, personality traits^103–105^, measures of intelligence^58,59,106,107^, and social traits related to autism^46,55^ including scores on the SCDC, as previous research has investigated the phenotypic correlation between these domains and systemising^10,78,108–112^.

To understand the correlation between systemising and various phenotypes that have been genetically correlated with autism, we used GWAS data from 15 phenotypes including autism. 10 of these phenotypes (cognitive aptitude^59^, educational attainment^58^, tiredness^113^, neuroticism^103^, subjective wellbeing^103^, schizophrenia^114^, major depression^102^, depressive symptoms^103^, ADHD^63^, and chronotype^115^), have been previously reported to be significantly genetically correlated with autism out of 234 phenotypes tested using LDHub^62^ (*P* < 2.1 × 10^−4^). We excluded college degree from this list, as previous work has identified near perfect genetic correlation between educational attainment and college degree^58^. In addition, we included data from friendship satisfaction^55^, family relationship satisfaction^55^, systemising, and self-reported empathy^46^, all of which are also significantly genetically correlated with autism with *P* < 2.1 × 10^−4^. These four additional phenotypes were not included in the previous paper which investigated genetic correlations with autism. Details of sample sizes with PMIDs/DOIs are provided in Supplementary Data 12. Cross trait genetic correlations were computed for all 15 phenotypes, and results were corrected for multiple testing using Bonferroni correction. A correlogram was created after using hierarchical clustering to cluster the phenotypes.

To investigate whether the combination of negative genetic correlation social traits and positive genetic correlation for non-social traits is specific to autism, we conducted a genetic correlation between all psychiatric conditions for which we had access to summary GWAS statistics (ADHD^63^, Anxiety^116^, Autism^57^, Anorexia^101^, Bipolar Disorder^99^, Major Depressive Disorder^102^, OCD^117,118^, PTSD^119^, and Schizophrenia^114^) and SQ-R, self-reported empathy measured using the EQ^46^ and friendship satisfaction^55^. We chose friendship satisfaction and self-reported empathy as representative of social traits as these are the most relevant to the social domain of autism for which we had access to GWAS summary statistics. The EQ is a short, 40-item self-report measure of empathy, which has been widely used and has good psychometric properties^60,120^. In addition, differences in aspects of empathy compared to the neurotypical population have been widely reported in autism^50,51,121^, and empathy is one of the items in measures such as ADOS-G. Friendship satisfaction was selected as difficulties in making friends is listed as a criteria for an autism diagnosis in the DSM-5^1^.

### GWIS and GSEM

To investigate whether the SQ-R is genetically correlated with autism independent of the genetic effects of educational attainment, we constructed a unique SQ-R phenotype after conditioning on the genetic effects of educational attainment using GWIS^122^. GWIS takes into account the genetic covariance between the two phenotypes to calculate the unique component of the phenotypes as a function of the genetic covariance and the ${{h}}_{{\mathrm{SNP}}}^2$. Before performing GWIS, we standardised the beta coefficients for the SQ-R GWAS by using the following formula:

$$\widehat {B_{{\mathrm{std}}}} = \hat B\sqrt {\frac{{2\left( {{\mathrm{MAF}}} \right)\left( {1 - {\mathrm{MAF}}} \right)}}{{\sigma _y^2}}},$$

where $\widehat {B_{{\mathrm{std}}}}$ is the standardised regression coefficients, $\hat B$ is the regression coefficient obtained from the non-standardised GWAS, MAF is the minor-allele frequency, $\sigma _y^2$ is the variance of the SQ-R. This equation is explained in detail in the Supplementary Note. We conducted GWIS using only educational attainment as we were unclear whether the GWAS of cognitive aptitude^59^ was conducted on a standardised phenotype. Further, there is a high genetic correlation between cognitive aptitude and educational attainment. In addition to GWIS, to validate the findings, we conducted GSEM^123^, a complementary but independent method. GSEM uses the genetic correlations and covariances calculated using LDSR after accounting for sample overlap.

### Polygenic scores in the SSC, AGRE, EU-AIMS LEAP, and Paris cohorts

We generated polygenic scores for SQ-R (mean weighted score of all the alleles that contribute to higher systemising) in 2221 probands from the SSC (Discovery dataset). We downloaded genotype data from the SSC from SFARI base (https://www.sfari.org/resource/sfari-base/). Individuals were genotyped on three different platforms: Illumina Omni2.5, Illumina 1Mv3, or Illumina 1Mv1. Informed consent or assent was obtained from all participants. In addition, the research team obtained ethical approval from the Cambridge Human Biology Research Ethics Committee to access and analyse the de-identified data from the SSC. We conducted stringent quality control and imputation separately for each platform. The full pipeline is available here: https://github.com/autism-research-centre/SSC_liftover_imputation. Briefly, individuals were excluded if they had: a genotyping rate < 95%, excessive or low heterozygosity (less or more than 3 SD from the mean), mismatched reported and genetic sex, and families with Mendelian errors > 5%. We further removed SNPs that significantly deviated from Hardy–Weinberg equilibrium (*P* < 1 × 10^−6^), had Mendelian errors in >10% of the families, and SNPs that were not genotyped in >10% of the families. We then conducted multi-dimensional scaling using the HapMap3 phase 3 population using the unrelated individuals CEU and TSI populations as representatives of the European population. This was conducted only in the parents to retain unrelated individuals for multi-dimensional scaling. Genetic principal components were calculated using only SNPs with minor-allele frequency > 5%, and pruning the SNPs in Plink using an *r*^2^ of 0.2. We excluded families from further downstream analyses if either one the parents were greater or less than 5 standard deviations from the means of the first two genetic principal components calculated using only the unrelated individuals in HapMap3 CEU and TSI populations. Quality control was done using Plink v 1.9 and R. Phasing and imputation were conducted using the Michigan Imputation Server (https://imputationserver.sph.umich.edu/start.html) using the 1000 genomes Phase 3 v5 as the reference panel.

Polygenic scores were generated using PRSice2 (https://choishingwan.github.io/PRSice/) for the SQ-R using the non-stratified GWAS data. We calculated the mean polygenic score for each of the 2221 probands in the SSC, after clumping SNPs using an *R*^2^ threshold of 0.1. Prior to generating polygenic scores, we confirmed that the probands were not related to each other using identity by descent PI-HAT > 0.15 as a relatedness cut-off. We used a *P*-value threshold of 1 as previous research on educational attainment, subjective wellbeing and social relationship satisfaction, all suggest that the maximum variance explained is at a threshold of 1 (refs. ^58,103^). This is expected for highly polygenic traits where many SNPs incrementally contribute to the variance explained^124^. Polygenic scoring was done using standardised scores on two different phenotypes as the dependent variable (RBS-R and the social and communication domain of the ADOS-G). We included sex, platform, the first 15 genetic principal components and standardised full-scale IQ as covariates. In addition, for the analysis of ADOS-G, we included the ADOS-G module as a covariate. Linear regression was conducted in R. A total of 135,233 SNPs were included in the polygenic score analyses after clumping and thresholding.

To validate the polygenic scores, we conducted additional polygenic score analysis using data combined from the AGRE, EU-AIMS LEAP and Paris cohorts. We followed similar quality control and imputation procedures to the SSC cohort. Given that this dataset was a mix of related and unrelated individuals, we chose unrelated individuals using a genomic relationship matrix (GRM) as provided in GCTA (grm-cutoff 0.05)^125^. To calculate GRMs, we included only SNPs with minor-allele frequency > 1%. Scripts are provided here: https://github.com/vwarrier/PARIS_LEAP_analysis. Polygenic scores were calculated using PRSice2 as described for the SSC data. Given the differences in dataset, polygenic scores were calculated separately for the AGRE dataset, and the EU-AIMS LEAP and Paris datasets combined. For each regression, we included sex and the first ten genetic principal components (standardised). The dependent variables were standardised scores on the RBS-R (*N* = 426) and the ADOS-G social and communication subscale (*N* = 475). IQ information was unavailable for most individuals, and hence we did not include IQ as a covariate. We combined the results of the EU-AIMS LEAP and Paris cohorts, and the AGRE dataset using inverse-variance weighted fixed-effect meta-analysis using the formula below:

$$\begin{array}{*{20}{l}} {w_i} \hfill & = \hfill & {1/{\mathrm{SE}}_i^2} \hfill \\ {{\mathrm{SE}}_{{\mathrm{meta}}}} \hfill & = \hfill & {\sqrt {1/{\mathrm{\Sigma }}_iw_i} } \hfill \\ {Beta_{{\mathrm{meta}}}} \hfill & = \hfill & {{\mathrm{\Sigma }}_i\beta _iw_i/{\mathrm{\Sigma }}_iw_i} \hfill \end{array},$$

where *β*_*i*_ is the standardised regression coefficient of the polygenic scores, SE_*i*_ is the associated standard error, and *w*_*i*_ is the weight.

### Bivariate GREML

We conducted bivariate genetic correlation using GCTA GREML to test the genetic correlation between the ADOS social and communication domains and the RBS-R scores. We created a GRM after including autistic individuals from the SSC, AGRE, EU-AIMS LEAP, and Paris cohorts. We excluded SNPs and individuals using the same quality control pipeline as applied to the SSC dataset outlined in the section above. We further restricted our analysis only to SNPs with a minor-allele frequency > 1%. We excluded related individuals (–grm-cutoff 0.05) resulting in a total of 2989 individuals. Of this, 2652 individuals had scores for the ADOS social and communication domain and 2550 individuals had scores on the RBS-R. We included sex and the first ten genetic principal components as covariates.

### Reporting summary

Further information on research design is available in the Nature Research Reporting Summary linked to this article.

## Supplementary information

Supplementary Information

Description of Additional Supplementary Items

Supplementary Data 1-13

Reporting Summary

## Data Availability

The SQ-R GWAS results are available from 23andMe. The full set of summary statistics can be made available to qualified investigators who enter into an agreement with 23andMe that protects participant confidentiality. Interested investigators should email dataset-request@23andme.com for more information. Top SNPs (*n* = 10,000) can be visualised here: https://ghfc.pasteur.fr.Data. for ALSPAC can be requested here: http://www.bristol.ac.uk/alspac/researchers/access/. Data from the Simons Simplex Collection can be requested here: https://www.sfari.org/resource/sfari-base/. Summary GWAS statistics were downloaded from the PGC consortium: http://www.med.unc.edu/pgc/results-and-downloads. Data for chronotype was downloaded from http://www.t2diabetesgenes.org/data/. Data for self-reported tiredness was downloaded from http://www.ccace.ed.ac.uk/node/335. Additional source data are available in Supplementary Data 1–13.

## Code availability

Genomic-SEM: https://github.com/MichelNivard/GenomicSEM, GWIS: https://sites.google.com/site/mgnivard/gwis, Plink: https://www.cog-genomics.org/plink2/, PRSice2: https://choishingwan.github.io/PRSice/, CAVIAR: http://genetics.cs.ucla.edu/caviar/, Michigan Imputation Server: https://imputationserver.sph.umich.edu/index.html, custom code for quality control of the SSC and the other cohorts can be downloaded from https://github.com/autism-research-centre/SSC_liftover_imputation (10.5281/zenodo.3342561) and from https://github.com/vwarrier/PARIS_LEAP_analysis (10.5281/zenodo.3342569).
