# Supplementary material for: Social and non-social autism symptoms and trait domains are genetically dissociable
Source: Commun Biol. 2019 Sep 3;2:328. doi: 10.1038/s42003-019-0558-4 (PMC6722082; doi:10.1038/s42003-019-0558-4)
Supplement: Supplementary file 2 — Description of Additional Supplementary Items [file 42003_2019_558_MOESM2_ESM.pdf]

Description of additional supplementary files

Supplementary Data 1-13: Excel file containing the following spreadsheets

Spreadsheet	Description
S1	Independent SNPs with P < 1E-6 from the GWAS studies
S2	MAGMA gene based analyses
S3	Effect direction concordance
S4	Additive heritability
S5	Variance explained by top SNPs
S6	LDSR Partitioned heritability (baseline categories)
S7	LDSR partitioned heritability (open chromatin regions)
S8	LDSR partitioned heritability (genes with brain-specific expression)
S9	Genetic correlations
S10	Genetic correlations - GWIS
S11	Genetic correlations between 15 phenotypes that are genetically correlated with autism
S12	Sample sizes and PMID of the 15 GWAS
S13	Genetic correlation between the SQ-R, empathy, friendship satisfaction, and 10 psychiatric conditions
