# Supplementary material for: Social and non-social autism symptoms and trait domains are genetically dissociable
Source: Commun Biol. 2019 Sep 3;2:328. doi: 10.1038/s42003-019-0558-4 (PMC6722082; doi:10.1038/s42003-019-0558-4)
Supplement: Supplementary file 4 — Reporting Summary [file 42003_2019_558_MOESM4_ESM.pdf]

nature research

Corresponding author(s): Warrier
Last updated by author(s): Jul 19, 2019

Reporting Summary

Nature Research wishes to improve the reproducibility of the work that we publish. This form provides structure for consistency and transparency in reporting. For further information on Nature Research policies, see Authors & Referees and the Editorial Policy Checklist.

Statistics

For all statistical analyses, confirm that the following items are present in the figure legend, table legend, main text, or Methods section.

n/a	Confirmed	
☐	☒	The exact sample size (n) for each experimental group/condition, given as a discrete number and unit of measurement
☐	☒	A statement on whether measurements were taken from distinct samples or whether the same sample was measured repeatedly
☐	☒	The statistical test(s) used AND whether they are one- or two-sided<br>*Only common tests should be described solely by name; describe more complex techniques in the Methods section.*
☐	☒	A description of all covariates tested
☐	☐	A description of any assumptions or corrections, such as tests of normality and adjustment for multiple comparisons
☐	☒	A full description of the statistical parameters including central tendency (e.g. means) or other basic estimates (e.g. regression coefficient) AND variation (e.g. standard deviation) or associated estimates of uncertainty (e.g. confidence intervals)
☐	☒	For null hypothesis testing, the test statistic (e.g. F, t, r) with confidence intervals, effect sizes, degrees of freedom and P value noted<br>*Give P values as exact values whenever suitable.*
☒	☐	For Bayesian analysis, information on the choice of priors and Markov chain Monte Carlo settings
☒	☐	For hierarchical and complex designs, identification of the appropriate level for tests and full reporting of outcomes
☐	☒	Estimates of effect sizes (e.g. Cohen's d, Pearson's r), indicating how they were calculated

Our web collection on statistics for biologists contains articles on many of the points above.

Software and code

Policy information about availability of computer code

Data collection	No specific software has been used to collect data. Please see the section below on how the data can be accessed. Please see the text box below on codes used to analyse the data.
Data analysis	Genomic-SEM: https://github.com/MichelNivard/GenomicSEM<br>GWIS: https://sites.google.com/site/mgnivard/gwis<br>Plink: https://www.cog-genomics.org/plink2/<br>PRSice2: https://choishingwan.github.io/PRSice/<br>CAVIAR: http://genetics.cs.ucla.edu/caviar/<br>Michigan Imputation Server: https://imputationserver.sph.umich.edu/index.html<br>Custom code for quality control of the SSC and the other cohorts can be downloaded from https://github.com/autism-research-centre/SSC_liftover_imputation<br>(DOI: 10.5281/zenodo.3342561) and from<br>https://github.com/vwarrier/PARIS_LEAP_analysis<br>(DOI: 10.5281/zenodo.3342569)

For manuscripts utilizing custom algorithms or software that are central to the research but not yet described in published literature, software must be made available to editors/reviewers. We strongly encourage code deposition in a community repository (e.g. GitHub). See the Nature Research guidelines for submitting code & software for further information.

Data

Policy information about availability of data

All manuscripts must include a data availability statement. This statement should provide the following information, where applicable:

- Accession codes, unique identifiers, or web links for publicly available datasets
- A list of figures that have associated raw data
- A description of any restrictions on data availability

The SQ-R GWAS results are available from 23andMe. The full set of summary statistics can be made available to qualified investigators who enter into an agreement with 23andMe that protects participant confidentiality. Interested investigators should email dataset-request@23andme.com for more information. Top SNPs (N = 10,000) can be visualized here: https://ghfc.pasteur.fr. Data for ALSPAC can be requested here: http://www.bristol.ac.uk/alspac/researchers/access/. Data from the Simons Simplex Collection can be requested here: https://www.sfari.org/resource/sfari-base/. Summary GWAS statistics were downloaded from the PGC consortium: http://www.med.unc.edu/pgc/results-and-downloads. Data for chronotype was downloaded from http://www.t2diabetesgenes.org/data/. Data for self-reported tiredness was downloaded from http://www.ccace.ed.ac.uk/node/335. Data from the Nijmegen Biomedical Study can be requested from Dr. Geert Poelmans (Geert.Poelmans@radboudumc.nl).

Field-specific reporting

Please select the one below that is the best fit for your research. If you are not sure, read the appropriate sections before making your selection.

☒ Life sciences ☐ Behavioural & social sciences ☐ Ecological, evolutionary & environmental sciences

For a reference copy of the document with all sections, see nature.com/documents/nr-reporting-summary-flat.pdf

Life sciences study design

All studies must disclose on these points even when the disclosure is negative.

Sample size

Given the limited availability of participants with the required phenotypic data, sample sizes included in the study varied considerably. The primary GWAS of the SQ-R had an N < 50,000. These included all participants from 23andMe who completed the SQ-R questionnaire and met the genetic QC criteria. Whilst this is still small for gene discovery, as mentioned in the manuscript, the sample size was sufficient for subsequent LDSC-based analyses. The sample sizes for the autistic data is small, but we included all data to our knowledge where phenotypic information was available on the two domains tested (social and non-social). We did not conduct any a priori power calculations.

Data exclusions

We excluded individuals and genetic data using standard QC procedures in GWAS studies. Briefly, unrelated participants were included if they had a call rate of greater than 98.5%, and were of primarily European ancestry (97% European ancestry). A total of 1,030,430 SNPs (including InDels) were genotyped. SNPs were excluded if: they failed the Hardy-Weinberg Equilibrium Test at P < 10-20; had a genotype rate of less than 90%; they failed the parent-offspring transmission test using trio data in the larger 23andMe research participant database; or if allele frequencies were significantly different from the European 1000 Genomes reference data (chi-square test, P < 10-20).

Replication

We conducted several analyses to validate the primary results.

First, we investigated and confirmed that the majority of the top independent SNPS (P < 1E-6) in the SQ-R GWAS had a concordant effect direction in related, genetically correlated GWAS of autism, educational attainment, cognitive aptitude, and insistence on sameness.

Second, using two methods (Genomic SEM and GWIS) and two datasets (GWASs of cognitive aptitude and educational attainment), we confirmed that the SQ-R is genetically correlated with the GWAS for autism independent of the genetic correlates of cognition/educational attainment.

To further validate the SQ-R GWAS, we tested if polygenic scores from the SQ-R GWAS are associated with restricted and repetitive behaviour in autistic individuals from the Simons Simplex Collection (N = 2,221). We confirmed that this was the case but did not observe an association with scores on the social and communication domain. We validated this using a smaller second dataset (N = 426 for restricted and repetitive behaviour and N = 475 for social and communication domain), and observed a concordance in effect direction. Meta-analysis of the discovery and the validation datasets improved the P-values of the initial association for the restricted and repetitive behaviour scale. In contrast, the association with the social and communication domain remained non-significant.

Using data from multiple different GWAS of social and non-social traits related to autism in the typical population, we identified limited genetic correlation between the social and non-social autistic traits. We validated this using a smaller sample of autistic individuals (N = 2,989), where we were unable to identify a significant genetic correlation between repetitive behaviour and the social and communication domain using bivariate GCTA-GREML.

Randomization

There was no randomization in this group as the analyses were quantitative trait GWAS/polygenic score analysis.

Blinding	No blinding was performed in this study.

Reporting for specific materials, systems and methods

We require information from authors about some types of materials, experimental systems and methods used in many studies. Here, indicate whether each material, system or method listed is relevant to your study. If you are not sure if a list item applies to your research, read the appropriate section before selecting a response.

Materials & experimental systems

n/a	Involved in the study
☒	☐ Antibodies
☒	☐ Eukaryotic cell lines
☒	☐ Palaeontology
☒	☐ Animals and other organisms
☐	☒ Human research participants
☒	☐ Clinical data

Methods

n/a	Involved in the study
☒	☐ ChIP-seq
☒	☐ Flow cytometry
☒	☐ MRI-based neuroimaging

Human research participants

Policy information about studies involving human research participants

Population characteristics

We have used several cohorts in this study. Three of these cohorts are from the general population. The primary cohort are individuals drawn from 23andMe, and we conducted a GWAS of the primary phenotype in this (SQ-R). A total of 51,564 participants completed the SQ-R (males = 26,063, and females = 25,501).

The second cohort from the typical population is ALSPAC, where we conducted a GWAS of the SCDC, measured at age 8 (N = 5,421).

Additionally, we also included data from 1,981 unrelated individuals (1000 males, 1981 females) from the Nijmegen Biomedical Study (NBS) to provide support for the independent SNPs with P < 1x10-6 in the non-stratified GWAS.

We additionally included 3 clinical cohort: SSC (N = 2,221), AGRE, and the EU-AIMS + PARIS cohorts.

Recruitment

23andMe: Research participants in the GWAS of the SQ-R were from 23andMe and are described in detail elsewhere(Ref:55 in the manuscript). All participants provided informed consent and answered surveys online according to a human subjects' research protocol, which was reviewed and approved by Ethical & Independent Review Services, an external AAHRPP-accredited private institutional review board (http://www.eandireview.com). All participants completed the online version of the SQ-R on the 23andMe participant portal.

ALSPAC: ALSPAC is a longitudinal cohort which recruited pregnant mothers in the Avon region of the UK. The ALSPAC cohort comprises 14,541 initial pregnancies from women in Avon resulting in a total of 13,988 children who were alive at 1 year of age. Children were enrolled in additional phases, described in greater detail elsewhere(Ref: 71 in the manuscript). This study received ethical approval from the ALSPAC Law-and-Ethics Committee, and the Cambridge Human Biology Research Ethics Committee.

Other cohorts: We included data from four cohorts to conduct polygenic score and bivariate genetic correlation analysis. The SSC (N = 2,221 unrelated autistic individuals) consists of simplex autistic families, and are described elsewhere(Ref: 72 in the manuscript). The AGRE cohort (N = 482 unrelated autistic individuals) consists of multiplex autism families, details of which are provided elsewhere(Ref: 73 in the manuscript). Across all cohorts, all participants were of European ancestry as identified using multi-dimensional scaling. Additionally, we included 401 individuals (including 25 neurotypical individuals) from the EU-AIMS LEAP(Ref: 74 in the manuscript) and Paris (Ref: 75 in the manuscript) cohorts. Across all cohorts, we included only unrelated individuals, who were predominantly of European Ancestry as defined by genetic principal components (5 SD deviations above or below the mean European PC1). Details of recruitment of the NBS cohort is provided elsewhere (Ref: 43).

Ethics oversight

23andMe: All participants provided informed consent and answered surveys online according to a human subjects' research protocol, which was reviewed and approved by Ethical & Independent Review Services, an external AAHRPP-accredited private institutional review board (http://www.eandireview.com).

ALSPAC: This study received ethical approval from the ALSPAC Law-and-Ethics Committee. Written informed consent was obtained from parent or a responsible legal guardian for the child to participate. Assent was obtained from the child participants where possible.

Other datasets: This study received ethical approval from the Cambridge Human Biology Research Ethics Committee to work with de-identified data.

Note that full information on the approval of the study protocol must also be provided in the manuscript.
